# Bridging Neuroscience and Clinical Practice: Accelerating Stroke Recovery in a Pontine Infarction Case Through Neuroplasticity-Based Rehabilitation

**DOI:** 10.7759/cureus.72563

**Published:** 2024-10-28

**Authors:** Wanli Lei

**Affiliations:** 1 Physical Therapy, College of Mathematics, Sciences, and Health Professions, Lincoln Memorial University, Knoxville, USA; 2 Rehabilitation, Knoxville Rehabilitation Hospital, Knoxville, USA

**Keywords:** foundational neuroscience, motor control tracts, neuroplasticity, pontine infarction, rehabilitation

## Abstract

Foundational neuroscience is crucial to locating lesions, understanding current functional limitations, making correct prognoses, and designing holistic and realistic treatment plans for stroke patients. A model bridging neuroscience knowledge and clinical practice was assessed through a rare pontine infarction case. A 76-year-old patient suffered two consecutive right-sided pontine ischemic strokes, leading to significant motor and sensory abnormalities on the left side. After the strokes stabilized, the patient was admitted to Knoxville Rehabilitation Hospital on August 10, 2023, under an intensive rehabilitation program grounded in neuroscience-based principles of plasticity. Remarkably, the patient fully recovered sensation by August 17. The clinical presentations, which included no ipsilateral cranial nerve dysfunction but the contralateral compromise of cranial nerves V and VII, indicated lesions in the right middle to caudal basilar pons. Employing available brainstem motor control systems, including reticulospinal and vestibulospinal tracts, the rehabilitation team designed a task-oriented, realistic neuroplasticity program to focus on gross motor skills such as postural control, bed mobility, transfers, walking, and stair negotiation, dynamically adjusted daily according to the patient’s progress, while waiting for the recovery of corticospinal tract. The patient successfully improved from varied levels of assistance to independently performing all tasks except stair negotiation, which required supervision due to safety concerns, possibly resulting from past silent cerebrovascular problems. The patient was discharged home on August 29 after demonstrating significant functional recovery. This study highlights leveraging foundational neuroscience knowledge to accelerate recovery and quickly improve stroke rehabilitation outcomes.

## Introduction

Stroke is a leading cause of disability worldwide, with many survivors experiencing lasting impairments that require structured rehabilitation to regain independence. The American Heart Association/American Stroke Association [1] and the U.S. Department of Veterans Affairs and Department of Defense [2] highlight the importance of timely, individualized, and multidisciplinary rehabilitation to address physical, cognitive, and psychological needs in stroke recovery. Both guidelines recommend early intervention, functional goal-setting, and integrating adaptive technologies to optimize outcomes, improve quality of life, and reduce healthcare costs through effective, sustained care [1,2].

Pontine stroke is less common but presents more severe functional loss, with hemorrhagic pontine stroke being worse (48.1% mortality) [3]. Structurally, the pons consist of a basilar component ventrally and a tegmental component dorsally. Functionally, the pons consists of the following three overlapping divisions: (A) serving as attachment locations for four pairs of cranial nerves (CNs) with their nuclei in the tegmental pons, including trigeminal nerve (CN V) in the mid-lateral pons, abducens (CN VI), facial (CN VII), and vestibulocochlear (CN VIII) nerves in the pontomedullary junction from medial to lateral. (B) Functioning as conduits for axon tracts exchanging information between the forebrain and midbrain to and from the medulla, cerebellum, and spinal cord. These axons group into descending tracts through the basilar pons and ascending tracts through the tegmental pons. (C) Engaging in integrative functions through other tracts, including motor control tracts from the brainstem, ascending reticular activating system, and cardiovascular and respiratory center pathways, among others, coursing through the tegmental pons [4].

Pontine ischemic stroke (infarction) only occurs in about 7% of brain strokes but 60~85% of brainstem strokes [5]. The pons gets blood supply from pontine arteries branching from the basilar artery in the paramedian component ventrally, from anterior inferior cerebellar artery (AICA) and superior cerebellar artery (SCA) in the posterolateral caudal and rostral tegmentum separately [6]. Still, the pons demonstrate huge vascular supply variations [5].

Stroke has become one of the seven significant contributors to chronic diseases in the United States, significantly impacting the economy. After a stroke, the nervous system initiates spontaneous, incomplete, and variable repair, known as neuroplasticity, through restructuring local and remote regions [7], either adaptive or maladaptive. Many traditional rehabilitation techniques have been under debate for their effectiveness and efficiency. However, intense task-specific activities are strongly recommended to guide adaptive functional neuroplasticity [1,2] in predictable critical timeframes, e.g., three months for motor functions, approximately five to six months for visuospatial/orientation functions, and months to years for cognitive functions [7], while patients can continue to gain functions after the critical periods [8]. Neurons are terminally differentiated cells, and the regeneration capacity of the nervous system is limited. Degrees of compensatory recovery post-stroke depend on the original severity of the injury.

Unilateral multiple pontine infarctions are the rarest type of pontine ischemic stroke with a poor prognosis [9]. This report employs such a rare double pontine infarction case to explore a model for an individualized rehabilitation program to manage stroke patients. Based on foundational neuroscience, a patient’s goal-directed, task-specific, activity-guided neuroplasticity rehabilitation program successfully shifted post-stroke supportive care to interventional management. It facilitated the patient to achieve his goals in 18 days.

## Case presentation

Patient

The patient is a 76-year-old male who initially presented with left lower extremity paresis and paresthesia on August 4, 2023. By August 5, his condition had worsened to involve the left side of his face and upper limb. A CT scan ruled out bleeding, and a subsequent diffusion-weighted MRI revealed two asymmetric areas of infarction in the right pons. Both imaging studies also noted a history of minor vascular injuries in the subventricular regions and the left thalamus of the brain. However, the patient was fully independent before the rare double right pontine infarctions. After stabilization with medications, he was discharged to Knoxville Rehabilitation Hospital on August 9, 2023, but showed no significant improvement in symptoms or functional ability.

Assessment

The patient received intensive rehabilitation from a multidisciplinary team, including physical, occupational, and speech-language pathologists. This report focuses solely on his physical therapy treatment. On August 10, the patient was alert and oriented to person, place, time, and situation (X4) and exhibited borderline hypotension (110/59 mmHg). He denied pain but complained of left-sided paresthesia. Clinically, he presented with dysphagia and dysarthria, unable to lift his left upper limb and walk. He had a left facial droop, but the forehead could still frown, in addition to normal tongue protrusion, intact eye movement without diplopia, and normal chewing functions. His primary goals were to “walk again” and “go home.”

The quality indicator (QI) system was used to assess the patient’s daily living activities, as shown in the admission column of Table 1. Functional levels were graded according to the assistance required: dependent or assistance of two, 1; substantial/maximal assistance, 2; partial/moderate assistance, 3; supervision, 4; set-up assistance, 5; independent, 6; not assessed due to a medical condition or safety concerns, 88.

**Table 1:** Qualitative index system for daily function assessment.

Quality indicator	Admission	Discharge
Roll left and right	3	6
Sit to lying	3	6
Lying to sitting on the side of the bed	2	6
Sit to stand	2	6
Chair/bed-to-chair transfer	2	6
Car transfer	2	6
Walk 10 feet	88	6
Walk 50 feet	88	4
Walk 150 feet	88	4
Walking 10 feet on uneven surfaces	88	4
Picking up object	88	4
1 step	88	6
Four steps	88	6
12 steps	88	4
Wheelchair 50 feet	2	No need
Wheelchair 150 feet	2	No need

The patient showed full strength of right lower limb muscles (5/5), but the left lower limb demonstrated general weakness (2-/5) in hip muscles and knee extensors and flaccidity in knee flexors and ankle dorsi/plantar flexors (0/5) (Table 2, Admission column).

**Table 2:** Muscle strength of the left lower limb.

Left lower extremity strength	Admission	Discharge
Hip		
Flexion	2-/5	5
Abduction	2-/5	5
Adduction	2-/5	5
Knee		
Flexion	0/5	5
Extension	2-/5	5
Ankle		
Plantar flexion	0/5	5
Dorsiflexion	0/5	5

Rehabilitation started on August 11 with three hours of physical, occupational, and speech-language treatment daily, five days a week. From admission to discharge, the patient denied pain. For physical therapy, each treatment started with a brief reassessment, followed by updated treatments focusing on the impaired functions, progressed with decreasing direct assistance, to verbal/tactile cues, to patient self-feedback without external cues, thus challenging the patient to gain compromised functions more effectively.

Bed Mobility Training

During bed mobility training, verbal cues, contact guard assistance, and minimal assistance to lift the lower left limb (QI score 3) were initiated. Body mechanics and hand placement to support the trunk were instructed to roll over in the bed and from side lying to sit up at the edge of the bed with trunk stability maintained.

Sit-to-Stand Transfer

Verbal cues, physical demonstration, contact guard assistance, and minimal assistance to support the trunk during transfer were initiated at the early stage (QI score 3). Body mechanics and hand placement on the assistive devices were instructed to improve the safety of sit-to/from-stand transfer, further exercised in parallel bars with bilateral handrail use, progressing to no handrail use, from mini squat to deep squat to mimicking the sitting process. The body shift to the right during squat exercise was corrected to maintain the body in the midline. Body mechanics and hand reaching out of a vision to grab the armrest during sit were practiced repeatedly until the safe transfer was achieved.

Gait Training

Gait training was more difficult for the patient due to the left foot drop in the early stage. Pre-gait exercises were performed early, including 2-inch cone stepping (over and side to side) and toe-tapping in parallel bars with bilateral handrail use. Verbal cues and physical demonstrations encouraged the patient to take more significant strides in an alternative pattern. Then, the patient started to walk with a front-wheeled walker. During the gait training, the left ankle was wrapped with an ACE bandage into dorsiflexion and eversion to facilitate floor clearance in a “step to” gait with moderate assistance to advance the left lower limb at the beginning (QI score 2). Left hip flexion and knee extension during the terminal swing phase were emphasized by verbal and tactile cues for floor clearance when the patient demonstrated increased strength of the left hip and knee. Gait training progressed to improve speed, endurance, and dynamic balance through walking longer distances over uneven and compliable surfaces with a faster pace in alternative steps.

Step/Stair Negotiation Training

The patient reported no stairs/steps at home during emergent hospitalization and discharge to the inpatient rehabilitation hospital; however, on August 17, the patient reported three steps to get into the house without handrails, and step/stair negotiation training was initiated. Verbal cues about bilateral hand placement on the rails, right foot leading during ascending, and left foot leading during descending with a “step-by-step” negotiation pattern were instructed and practiced. At the early training, contact guard assistance was provided with occasional minimal aid to advance the left lower limb and support the trunk (QI score 3).

Strengthening and Other Interventions

Considering the “shock” phase after the stroke, the transient “weakness” was unrelated to muscles. Thus, no specific strengthening exercises were performed but the task-specific activities mentioned above were focused upon. However, the strength of the left lower limb was screened on and after August 17 when increased active left hip flexion with decreased hip hike was noticed during gait training, followed by left hemisphere sensory assessment and bilateral lower limb quadriceps stretch (knee jerk) reflex and plantar flexor muscle stretch (plantar jerk) reflex assessment. Left hip extension/flexion, knee extension, and foot dorsiflexion were instructed as “home” exercises for the patient.

Outcomes

As shown in the discharge column of Table 1, all the goals of functional mobilities were achieved but not shown at different time points, reflecting the progress of task-specific, activity-guided neuroplasticity for recovery.

Bed Mobility

The patient started bed mobility training on August 11 with minimal assistance (QI score 3) and achieved independent bed roll and side-lying to sit (QI score 6) on August 15.

Sit-to/From-Stand Transfer

The patient started sit-to/from-stand transfer training on August 11 with minimal assistance (QI score 3) and became fully independent on August 15. The patient was more compliant after gaining back such daily functions in the short term. However, for safety reasons and to avoid falls in the rehabilitation facility, therapists and other healthcare providers supervised the transfer until discharge on August 29 (QI score 6).

Gait Training

The patient started walking with moderate assistance (QI score 2) on August 11 to supervision on August 17 (QI score 4) with continued improvement in speed, endurance, and dynamic balance with abnormal compensated steppage gait, mainly showing decreased left hip and knee extension during the pre-swing phase without foot pushup, as well as decreased hip flexion and knee extension without ankle dorsiflexion during terminal swing. The patient demonstrated a “steppage gait” with decreased hip hike during walking since August 17 after stopping the ACE wrap, and the patient did not show gait pattern recovery further. Repeated verbal and tactile cues were used during gait training to increase the left hip flexion and knee extension for foot clearance. Still, there was no further improvement after August 25. The patient showed better foot clearance for about 10 steps after each cue, demonstrating a short attentional span; then, the left sole was dragged on the floor. However, no left foot drop was observed after August 17 without ACE wrapping, especially in the terminal swing phase, with the ankle in a neutral position, although the ankle dorsiflexor strength was assessed as 0/5.

Step/Stair Negotiation

The patient started step/stair negotiation training with moderate assistance (QI score 3) on August 17 and improved well to negotiate 12 6-inch steps under supervision at discharge (QI score 4). There was no difficulty with 1 and 4 steps (QI score 6). Still, the patient could not remember the foot sequencing during ascending and descending stairs or missed the step-by-step pattern, resulting in frequent loss of balance when negotiating 12 steps.

Left Lower Limb Strength

Although no specific strengthening exercises were performed, the patient showed autonomously improved left hip flexion on August 17; since then, left lower limb muscle strength has been screened frequently. The patient demonstrated difficulty with left hip flexion and knee extension in sitting; however, if the left hip or knee were passively positioned into flexion or extension separately, the patient demonstrated 3+/5 strength. The left ankle was still lax, and the patient could not hold the foot after being passively positioned into neutral or dorsiflexion. When the patient stood up in the parallel bars, even with bilateral hands supported on the handrails to maintain trunk stability, he could not keep the passively flexed left hip or extended left knee in position. On August 22, the patient still demonstrated difficulty in performing left hip flexion and knee extension voluntarily in sitting but showed 5/5 strength when passively positioning the joints into flexion or extension, and the patient started to maintain passively flexed left hip and extended left knee when standing in the parallel bars with trunk stability maintained by hands over handrails. The left ankle showed minimal active dorsiflexion when sitting. The patient received one electrical stimulation treatment on the left tibialis anterior on August 24, and since then, all left lower limb muscles have been screened 5/5. Still, there was no change in the “steppage gait” pattern until discharge.

Other Assessments

On August 17, light touch was assessed along the left lower limb, left hand, and left face, and the patient reported normal sensation. On August 17, the patient demonstrated right knee jerk hyperreflexia (4+) and normal plantar jerk reflex (2+), with areflexia of the left knee and plantar jerk reflex (0). On August 25, the patient demonstrated bilateral knee jerk hyperreflexia (4+), normal right plantar jerk reflex (2+), and areflexia of left plantar jerk reflex (0), with no change until discharge.

## Discussion

Diffusion-weighted images can locate the stroke lesions but struggle to differentiate the lesional core from the penumbra region and correlate the injury with impaired functions [10]. Fine mapping is critical to designing a holistic and realistic rehabilitation program for patients with stroke, who usually only complain of the most compromised somatosensory and motor functions. This case documented the impaired functions and recovery time in detail to narrow down the lesional core of the two isolated pontine infarctions and update the daily treatment plans based on foundational neuroscience.

Although unilateral multiple pontine infarctions are rare and generally have poor prognoses for motor and sensory recovery [9], this case with two asymmetric foci in the right pons showed excellent recovery with sensory spared, indicating each stroke case must be treated individually. The two infarctions matched the patient’s clinical presentations on August 4 and 5 separately as left hemisphere paresthesia and paresis, indicating that both lesions affected the basilar and tegmental pons. According to the topographic mapping of motor functions in basilar pons [11], the patient suffered pontine infarction more laterally on August 4 and medially on August 5. Regular light touch was regulated, indicating that tegmental pontine impairment was related to penumbra effects or resolved by partially overlapping blood supply from AICA [4].

The infarction locations were further analyzed according to impaired motor functions of contralateral CN VII and XII [12]. The patient presented a left-sided facial droop with the forehead and scalp spared, which indicated that the corticobulbar tract was impaired before decussation to innervate the left CN VII motor nucleus on August 5. Therefore, both infarction lesions were in the paramedian but between the middle and the caudal basilar pons. The dorsal nucleus of CN VII has bilateral cingulate gyrus upper motor control and is spared, with forehead and scalp functions normal [13]. The left CN XII was expected to be affected by the right pontine infarction; however, the patient showed normal tongue protrusion without deviation to the left, indicating that the two pontine infarctions spared the corticobulbar tract that controls the CN XII. Despite bilateral corticobulbar tract control of CN IX, CN X, and CN XI, the target muscles of these three cranial nerves were compromised, as shown by dysphagia and dysarthria. The possible reason is that bilateral control still differentiates from mono- and disynaptic projections related to fine motor control.

The pons function as a conduit for the corticospinal tract (CST) in its basilar component. The other motor control tracts initiated in the tegmental pons, including the reticulospinal tract (RST) and vestibulospinal tract (VST), were further analyzed [4]. The CST consists of two types of axons. Axons from monosynaptic neurons in the caudal primary motor cortex (M1) develop postnatally and innervate the lower motor neurons in the ventral horn of the spinal cord directly and function in fine motor control and new skill learning [14]. Axons from disynaptic neurons in rostral M1 and other cortical regions develop prenatally for patterns of activities such as walking [14]. RST (both pontine and medullary) and VST (both lateral and medial) are modulated mainly by bilateral corticobulbar tracts with potential functional recovery for postural and gross motor control covering the trunk and proximal joints of extremities after stroke [15].

The patient showed proximal to distal recovery of strength and gross motor functions during rehabilitation, including sitting postural control, bed mobility, and sit-to/from-stand transfer in four days of rehabilitation, indicating that with the elapsed “shock” phase, the RST and VST started to function for motor control of posture and proximal extremities. While the CST for fine motor control and gait lagged, indicating patterns of activities and muscle strength are not directly related. Recent research showed that fine motor control of finger movement partially depended on strength recovery (~60% of pre-injury level); extra fine motor control, such as finger individuation movement, was independent of strength [16]. Therefore, task-specific activity training should be designed during post-stroke rehabilitation, not individual muscle strengthening.

Except for the fine motor control of the left lower limb, the patient also presented lagging fine motor control recovery related to tongue and hand functions. This pattern highly supported the most recently expounded integrate-isolate model of the M1 [17]. There are three disrupted concentric presentations of the body regions centered around the tongue, fingers, and toes, spaced by three integrative regions for whole-body control. Where the body parts need more fine motor control, there is less presentation along the M1; thus, less redundancy is available for compensation after injury.

Pontine infarction can also compromise cognitive capacities due to cerebrocerebellar functional loss [18]. The past medical history of silent left basal nuclei lacunar stroke and degenerated periventricular and subcortical white matter, as detected by the CT and MRI scan, can compromise cognition [19]. The degenerated periventricular zone can also delay corticobulbar, corticopontine, and CST recovery due to the loss of precursors of oligodendrocytes [20]. All these factors contributed to the slow recovery of motor functions, especially the fine motor control and gait related to CST tracts.

In this case, the rehabilitation program focused on RST and VST for the patient’s posture and gross motor control. It progressed correspondingly to the recovery of these tracts, successfully improving the patient’s daily functions quickly. Unfortunately, past medical history and current injuries delayed the recovery of the CST. The patient must continue skilled therapy to check if the CST can continue to recover while improving and maintaining the functions that have been gained.

## Conclusions

In summary, the patient achieved independence in bed mobility and sit-to/from-stand transfers by August 15, progressed in gait training with a compensated steppage pattern, and improved stair negotiation abilities by discharge. Strength limitations and short attentional spans affected specific motor tasks, with no further gait pattern improvement noted.

In this post-pontine stroke case, the author emphasizes that a strong foundation in neuroscience is essential for clinical reasoning and practice. This understanding enables clinicians to help stroke patients regain function as efficiently as possible, particularly given current medical resource limitations. The author has been applying these strategies to treat stroke cases and continues to refine this approach.

Future research should optimize these strategies and explore innovative therapies to enhance motor recovery in this challenging patient population. By integrating neuroanatomy, motor control, and neuroplasticity insights, rehabilitation professionals can create more effective, individualized interventions.
